# Changes in Hysterectomy Route and Adnexal Removal for Benign Disease in Australia 2001–2015: A National Population-Based Study

**DOI:** 10.1155/2018/5828071

**Published:** 2018-05-31

**Authors:** Natalie De Cure, Stephen J. Robson

**Affiliations:** ^1^Centenary Hospital for Women and Children, P.O. Box 11, Woden, ACT 2606, Australia; ^2^Department of Obstetrics and Gynaecology, Australian National University Medical School, P.O. Box 5235, Garran, ACT 2605, Australia

## Abstract

**Objective:**

Hysterectomy rates have fallen over recent years and there remains debate whether salpingectomy should be performed to reduce the lifetime risk of ovarian cancer. We examined trends in adnexal removal and route of hysterectomy in Australia between 2001 and 2015.

**Methods:**

Data were obtained from the national procedural dataset for hysterectomy approach (vaginal, VH; abdominal, AH; and, laparoscopic, LH) and rates of adnexal removal, as well as endometrial ablation. The total female population in two age groups (“younger age group,” 35 to 54 years, and “older age group,” 55 to 74 years) was obtained from the Australian Bureau of Statistics.

**Results:**

The rate of hysterectomy fell in both younger (61.7 versus 45.2/10000/year, *p* < 0.005) and older (38.8 versus 33.2/10000/year, *p* < 0.005) age groups. In both age groups there were significant decreases in the incidence rates for VH (by 53% in the younger age group and 29% in the older age group) and AH (by 53% and 55%, respectively). The rates of LH increased by 153% in the younger age group and 307% in the older age group. Overall, the proportion of hysterectomies involving adnexal removal increased (31% versus 65% in the younger age group, *p* < 0.005; 44% versus 58% in the older age group, *p* < 0.005). The increase occurred almost entirely after 2011.

**Conclusion:**

Hysterectomy is becoming less common, and both vaginal and abdominal hysterectomy are being replaced by laparoscopic hysterectomy. Removal of the adnexae is now more common in younger women.

## 1. Introduction

Hysterectomy for benign conditions remains a common procedure in Australia and internationally. Prior to 2000, the lifetime risk of hysterectomy in Australian women was estimated to be approximately 35% [1], similar to the rate in other developed countries [2, 3]. Since 2000—associated with the introduction of treatments such as the levonorgestrel-releasing intrauterine system (Mirena™) and second-generation endometrial ablation techniques—the rate of hysterectomy has fallen [4]. Over the same period nonsurgical treatments for fibroids such as uterine artery embolization and focussed ultrasound also might be expected to reduce further the rate of hysterectomy [5, 6].

A trend to decreased use of hysterectomy has been identified in some European countries and in North America [7, 8]. As treatments for heavy menstrual bleeding (HMB) have evolved so has the surgical approach to hysterectomy. Updating previous evidence [9, 10] a recent systematic review comparing total laparoscopic hysterectomy (TLH) with vaginal hysterectomy (VH) included 24 studies published to February 2016 and reported no difference between the two techniques in the rate of major or minor complications, risk of ureter and bladder injuries, intraoperative blood loss, and length of hospital stay [11]. VH was associated with a shorter operative time and lower rates of vaginal cuff dehiscence and conversion to laparotomy. However vaginal access and uterine size impose limitations on uptake of vaginal hysterectomy and there is a body of evidence supporting a laparoscopic approach as more appropriate where a vaginal approach is difficult [9, 10].

Further complicating the change in surgical paradigm is an evolving body of evidence that cells of the fallopian tube may be the origin of many high-grade serous ovarian tumours [12]. For this reason some professional societies now recommend discussion of opportunistic salpingectomy at the time of hysterectomy for benign conditions to reduce this risk [13, 14]. A systematic review of opportunistic salpingectomy identified ten comparative studies that cumulatively demonstrated a small to no increase in operative time and no additional blood loss, hospital stay, or complications attributable to salpingectomy at the time of hysterectomy for benign disease [15]. However, salpingectomy can be more challenging at VH and this has a potential to influence the choice of approach for hysterectomy [16]. We set out to determine how these factors might have influenced hysterectomy in Australia over the last 15 years. We wanted to determine if there had been a significant change in the incidence rate of hysterectomy; whether there had been changes in the approach to hysterectomy; and whether there has been an increasing trend to removal of the adnexae.

## 2. Materials and Methods

Data regarding hysterectomy and endometrial ablation were obtained from the Australian Institute of Health and Welfare (AIHW) national procedural database. The AIHW national procedural database holds information collected through the National Health Information Agreement as required by and specified in the National Minimum Data Set relating to hospitals. The data are supplied by all Australian state and territory health authorities. Procedures use an agreed national standard, the* Australian Classification of Health Interventions* (ACHI), which is based around the Australian National Medical Benefits Schedule (MBS). Validation studies of the AIHW dataset have reported 99.5% agreement with “true” morbidity in a female population (kappa 0.86) [17]. We selected data from 2001 to 2015 using procedures coded according to the ICD-10-AM/ACHI guidelines, as detailed in Box 1. Data were extracted only for benign disease: hysterectomy code numbers specific for malignant disease were identified but not extracted and were not included in the study dataset.

To provide a denominator, annual point estimates for the total female population in two age bands—35 to 54 years (the “younger age group”) and 55 to 74 years (the “older age group”)—were obtained from the Australian Bureau of Statistics (ABS). All extracted data were entered into Excel™ spreadsheets and statistical analysis was performed in GenStat (https://www.vsni.co.uk/software/genstat/). Polynomial linear regressions were performed to calculate the coefficient of determination (*R*^2^ values) as measures of the closeness of fit and *p* values. The study received prospective approval from the Human Research Ethics Committee of the Australian National University (protocol 2015/347).

## 3. Results

**Figure 1:**
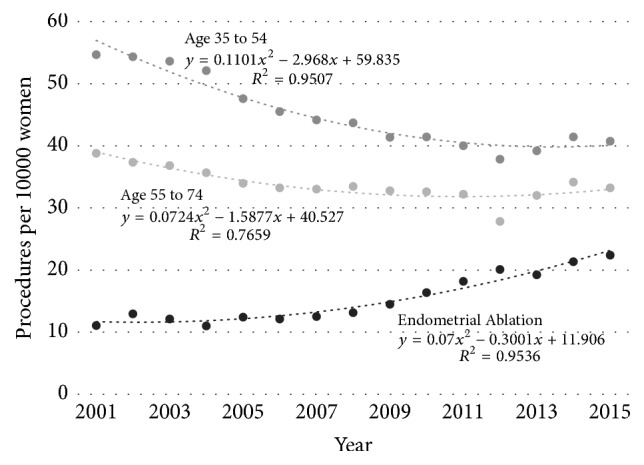
Age-stratified incidence rates of hysterectomy in Australia (procedures per 10000 women) for women aged 35–54 years and 55–74 years and incidence rate of endometrial ablation in women aged 35–54 years.

The overall incidence rate of hysterectomy at a national level fell (from 54.7 to 40.7/10000/year, *p* < 0.005) and this change occurred in both the younger (from 61.7 to 45.2/10000/year, *p* < 0.005) and older (from 38.8 to 33.2/10000/year, *p* < 0.005) age groups over the study period (Figure 1). Over the same time period the rate of endometrial ablation in the younger age group increased from 11.0 to 22.4/10000/year (*p* < 0.005).

**Figure 2:**
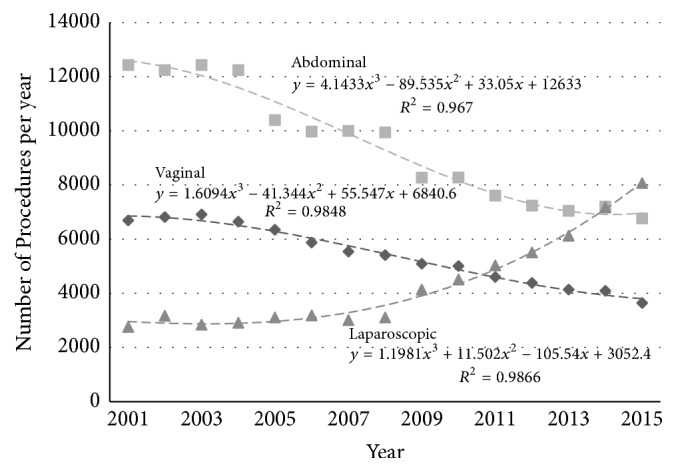
Absolute number of hysterectomies by different routes (◆ vaginal, ■ abdominal, and ▲ laparoscopic) in Australia in women aged 35–54 years.

**Figure 3:**
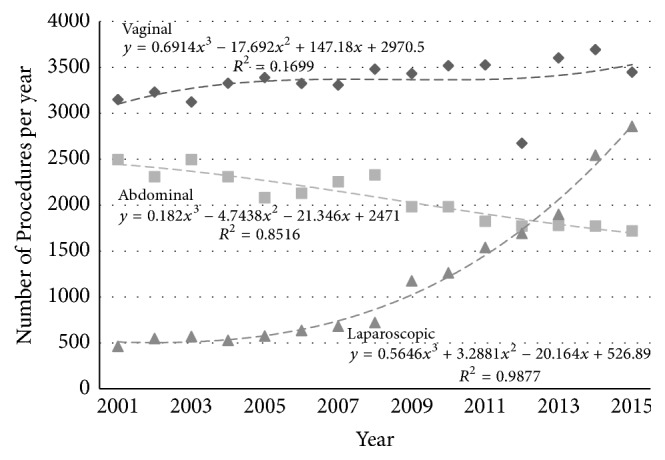
Absolute number of hysterectomies by different routes (◆ vaginal, ■ abdominal, and ▲ laparoscopic) in Australia in women aged 55–74 years.

The total number of hysterectomies performed in Australia by each route is presented in Figure 2 (the younger age group) and Figure 3 (the older age group).

**Figure 4:**
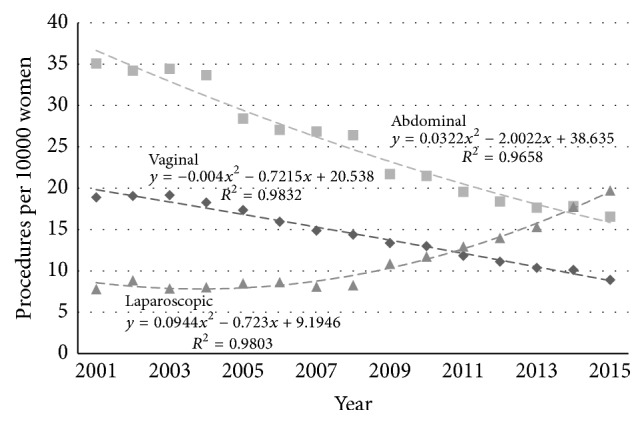
Age-stratified incidence rates for different routes of hysterectomy (◆ vaginal, ■ abdominal, and ▲ laparoscopic) in Australia (procedures per 10000 women) for women aged 35–54 years.

**Figure 5:**
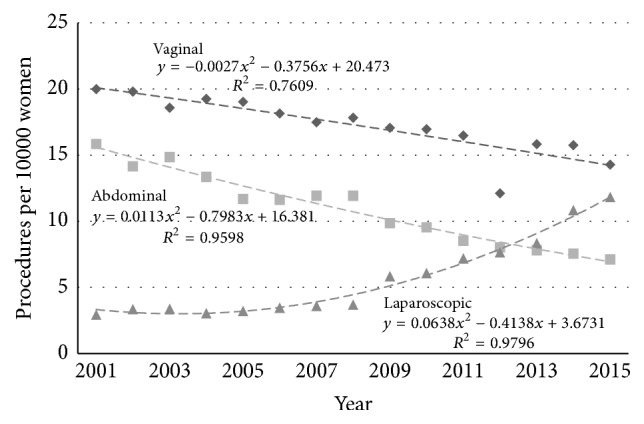
Age-stratified incidence rates for different routes of hysterectomy (◆ vaginal, ■ abdominal, and ▲ laparoscopic) in Australia (procedures per 10000 women) for women aged 55–74 years.

The incidence rates for the individual routes of hysterectomy (vaginal, abdominal, and laparoscopic) also changed in both age groups. In the younger age group (Figure 4) the rates fell for VH by 53% (from 18.9 to 8.9/10000/year, *p* < 0.005) and for AH also by 53% (from 35.1 to 16.5/10000/year, *p* < 0.005), while that of LH increased by 153% (from 7.8 to 19.7/10000/year, *p* < 0.005). In the older age group (Figure 5) the rate of VH fell by 29% (from 20.0 to 14.3/10000/year, *p* < 0.005) and for AH by 55% (from 15.9 to 7.1/10000/year, *p* < 0.005). The rate of LH increased by 307% (from 2.9 to 11.8/10000/year, *p* < 0.005).

**Figure 6:**
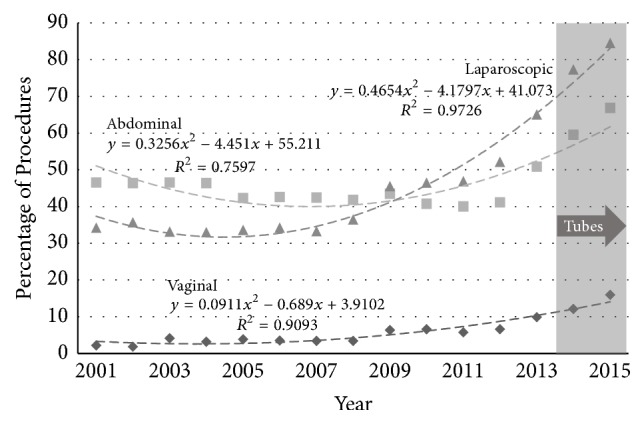
Percentage of procedures involving removal of the adnexae by route of hysterectomy (◆ vaginal, ■ abdominal, and ▲ laparoscopic) in Australia for women aged 35–54 years. The epoch in which guidance advised opportunistic salpingectomy is shaded.

**Figure 7:**
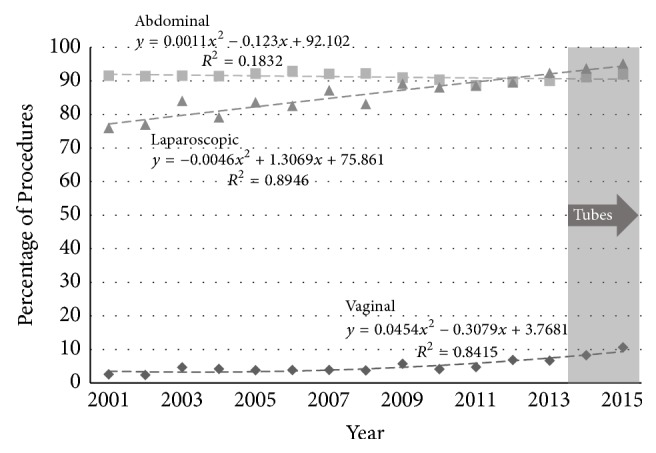
Percentage of procedures involving removal of the adnexae by route of hysterectomy (◆ vaginal, ■ abdominal, and ▲ laparoscopic) in Australia for women aged 55–74 years. The epoch in which guidance advised opportunistic salpingectomy is shaded.

The proportion of hysterectomies involving removal of adnexal structures increased significantly over the study period in the younger age group for each route of hysterectomy (Figure 6): by 623% in VH (from 2.2 to 15.9%, *p* < 0.005); by 44% in AH (from 46.6% to 66.9%, *p* = 0.09); and by 50% in LH (from 34.2% to 84.5%, *p* = 0.012). In the older age group (Figure 7) for AH the rate of adnexal removal was high and remained unchanged (from 91.5% to 91.9%, *p* = 0.11). The rate in LH increased from 76% to 95% (*p* < 0.005) and in VH the rate was low but increased from 2.6% to 10.6% (*p* < 0.005).

**Figure 8:**
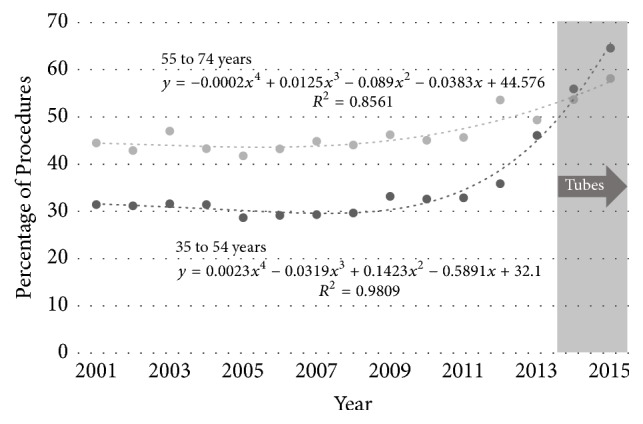
Percentage of all hysterectomies involving removal of the adnexae by age group (35 to 54 years, 55 to 74 years) for the period 2001 to 2015 in Australia. The epoch in which guidance advised possible opportunistic salpingectomy is shaded.

For hysterectomy by all approaches the proportion performed with associated adnexal removal has increased in both the younger (from 31% to 65%, *p* < 0.005) and older (from 44% to 58%, *p* < 0.005) (Figure 8). However, this increase has occurred almost entirely after 2011: there was no significant increase in the rate of adnexal removal from 2001 to 2011 in either the younger (*p* = 0.41) or older (*p* = 0.32) age groups.

## 4. Discussion

The findings of this study are consistent with published data from Europe and North America that have shown that hysterectomy is being undertaken less commonly in developed countries [2, 3]. Despite a weight of evidence supporting a vaginal approach [9, 10], the proportion of hysterectomies performed vaginally has fallen overall, with the greatest decrease has been seen in the younger age group. Importantly, the proportion of hysterectomies performed with associated adnexal removal has increased in the younger age group, but across both age groups there is a low rate with VH.

The findings of our study provide a comparison to other similar countries. A study from Denmark using data from a 35-year period until 2011 revealed that although there was considerable local variation, there had been only a small reduction in the overall rate of hysterectomy [7]. That study also revealed a trend away from abdominal surgery with an increased uptake of laparoscopic approaches, but no change in the rate of vaginal hysterectomy since 2003. In the United States, where the rate of hysterectomy has also fallen, the initial uptake of LH was slow [8], but there now has been acceleration in the use of laparoscopic and robotic hysterectomy [18].

The increase in removal of adnexal structures noted in this study mirrors a similar trend noted in the United States. The change is likely to reflect the evolving literature describing a clear association between dysplastic changes occurring in the distal fallopian and their relationship to ovarian malignancy [12, 19]. Our study showed low rates of adnexal removal associated with VH in younger women. The technical challenges in performing adnexal surgery at the time of VH are well-recognised, with authors commenting that with “the decreasing rate of … [vaginal hysterectomy]… the vaginal approach and [the added] complexity of a salpingectomy may make this approach seem less appealing” [17]. A population-based study from Sweden reported that women who had undergone salpingectomy during hysterectomy for benign disease had a decrease in subsequent risk for ovarian cancer with a hazard ratio of 0.65 and that women undergoing bilateral salpingectomy had 50% lower risk than those undergoing unilateral salpingectomy [12]. Those authors concluded that removal of the fallopian tubes is an effective measure to reduce ovarian cancer risk in the general population.

While systematic reviews continue to report that the VH is preferable for hysterectomy in benign disease, the ideal route for women unsuitable for a vaginal approach remains to be determined [9]. Meta-analysis of published randomised controlled trials favours LH but with the trade-off of a longer operating time [20]. Despite evidence that VH is associated with the best outcome, the use of VH has fallen. The Cochrane review group concluded that VH should be performed where possible, but where VH is not considered possible, LH may have advantages over AH. However, the length of the surgery increases as the extent of the surgery performed laparoscopically increases.

The trend to an increasing prevalence of obesity in developed countries is likely to affect both the operating time and the rate of complications associated with LH. Women who are obese have an increased risk of developing gynaecological conditions such as endometrial hyperplasia and heavy menstrual bleeding, making them more likely to require hysterectomy [21]. Over the period of our study the proportion of women with a body mass index (BMI) of 30 Kg/m^2^ in Australia was estimated to have increased by more than 13%, up to a prevalence of 55.9% [22]. Women with a high body mass index (BMI) are likely to be overrepresented in the hysterectomy group, and their operations are likely to use more operative time. A high BMI increases the duration of abdominal hysterectomy [23], and even after adjustment for patient age, parity, history of open surgery, previous caesarean section, and menopausal status, a significantly longer operating time—as much as doubling—was noted in the case of obese patients [24]. Indeed, the operating time for LH increases almost linearly with increasing BMI [25].

There are two important limitations to this study. Firstly, it is not possible to determine background rates of preexisting hysterectomy, so the population incidence rates reported are for women irrespective of whether they have a uterus or not. The age-related likelihood that a woman has already undergone hysterectomy is obviously cumulative, so the incidence rates we have reported underestimate the true rate of hysterectomy in women eligible for the procedure, that is, women who still have a uterus. The second limitation is that coding in the national dataset reflects nonspecific data regarding whether “removal of adnexal structures” was undertaken, and we were not specifically able to determine whether either isolated salpingectomy or salpingo-oophorectomy was performed at the hysterectomy. However, it seems likely that this change in the younger age group reflects salpingectomy alone since Australian guidance is explicit in discouraging oophorectomy before women reach their 1960s [14].

## 5. Conclusion

This study has confirmed the findings of other international studies that hysterectomy is becoming less common [7, 8] and that both vaginal and abdominal hysterectomy are being replaced by laparoscopic hysterectomy. At the same time, removal of the adnexae at the time of hysterectomy is now becoming more common in younger women.

## Figures and Tables

**Box 1:**
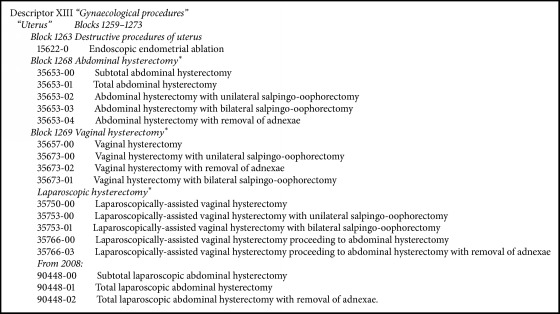
Search strategy. ^*∗*^Only descriptors for benign disease extracted.

## Data Availability

The data used to support the findings of this study are available from the corresponding author upon request.
